# Are Synbiotics added to the Standard Therapy to eradicate *Helicobacter pylori* in Children Beneficial? A Randomized Controlled Study

**DOI:** 10.5005/jp-journals-10018-1205

**Published:** 2017-05-05

**Authors:** Banu N Şirvan, Merve K Usta, Nurav U Kizilkan, Nafive Urganci

**Affiliations:** 1 Department of Pediatrics, Gaziosmanpasa Taksim Education and Research Hospital, Istanbul, Turkey; 2 Department of Pediatric Gastroenterology, Şişli Hamidiye Etfal Education and Research Hospital, Istanbul, Turkey; 3 Department of Pediatric Gastroenterology Koç University, Istanbul, Turkey

**Keywords:** Bifidobacterium lactis, *He/icobacter py/ori*, Synbiotic.

## Abstract

**Aim:**

We aimed to evaluate the role of the addition of *Bifidobacterium* /actis-containing synbiotic to the triple therapy in the case of *He/icobacter py/ori* eradication, the dyspeptic symptoms, and reducing the side effects of antibiotics.

**Materials and methods:**

A total of 104 children aged between 5 and 17 years, who were histopathologically diagnosed with *H. py/ori* were enrolled in this study, of whom 100 were included in the analysis. Patients were randomly classified into two groups. In the first group, 50 patients were administered amoxicillin + clarithromycin + lansoprazole for 14 days and *B.* /actis-containing synbiotic. In the second group, 50 patients were treated with the standard triple therapy. All patients were given information after completion of therapy.

**Results:**

*H. py/ori* eradication was achieved in 88% in group I who received standard therapy with additional synbiotic and 72% in group II (p = 0.046). The number of patients in the second group who suffered from abdominal pain between the 3rd and 14th day of the treatment was higher (p < 0.05). The addition of probiotics to the triple therapy significantly reduced the frequency of diarrhea, but no significant difference was detected in the frequency of metallic taste (p = 0.04, p = 0.418 respectively).

**Conclusion:**

The addition of synbiotic to the triple therapy is effective for eradicating *H. py/ori* infection in children and is usually helpful to reduce or eliminate dyspeptic symptoms like abdominal pain, diarrhea, and vomiting. This study suggest that improved tolerance to the eradication treatment also reduces the treatment failure by adding probiotics and encourages the future study using probiotic supplementation in *H. py/ori* treatment.

**How to cite this article:** Şirvan BN, Usta MK, Kizilkan NU, Urganci N. Are Synbiotics added to the Standard Therapy to eradicate *He/icobacter Py/ori* in Children Beneficial? A Randomized Controlled Study. Euroasian J Hepato-Gastroenterol 2017;7(1):17-22.

## INTRODUCTION

*Helicobacter pylori* is an infection common in developed and developing countries; it is thought to infect 50% of the population worldwide, it is acquired in childhood, and it is intended to be treated for gastric and duodenal ulcer, gastric cancer, atrophic gastritis, mucosa-associated lymphoid tissue lymphoma, and its other complica-tions.^1-3^ Recommendations for *H. pylori* in children were given in 2011, which included proton pump inhibitors (PPIs) + amoxicillin + imidazole or PPI + amoxicillin + clarithromycin or bismuth salts + amoxicillin + imidazole or sequential therapies for 7 to 14 days as first-line eradication treatment options.^4^ Failures have been observed, especially in childhood, despite the recommended treatment regimens.^5,6^ The major causes of failure are noncompliance of the patients and antibiotic resistance due to overuse or misuse.^7^

Probiotics are live microorganisms that may confer health benefits on the host when given in appropriate amounts.^8^ In recent years, many studies have been performed and meta-analyses have been published on probiotics, especially in adults, due to the failure observed in the eradication treatment of *H. pylori.* While studies on children are more limited, the key messages of the review by Szajewska et al,^9^ which covered last 5 years, stated that there is evidence supporting the use of probiotics in the treatment of several diseases, such as *H. pylori infection,* which also indicated the need for further studies to identify the strains, to determine the amounts, and to investigate the safety.

In the literature, studies on addition of probiotics to *H. pylori* eradication treatment in children are mostly related to *Lactobacilli [L. rhamnosus GG* (LGG), *L. johnso-nii, L. acidophilus, L. gasseri]* and *Saccharomyces boulardii.^10^*Studies with synbiotics in this respect are more limited. We aimed to investigate the additional favorable or unfavorable effects of a synbiotic *(Bifidobacterium lactis + inulin)* added to the *H. pylori* eradication treatment on the success of and during the treatment.

## MATERIALS AND METHODS

The patient population in our study consisted of 104 patients aged between 5 and 17 years who had presented with the complaints of chronic or recurrent abdominal pain and dyspepsia, had required endoscopic examination, had undergone upper gastrointestinal system (GIS) endoscopy, and was histopathologically diagnosed with *H. pylori* through biopsy. The study was conducted during the 1-year period between August 2013 and August 2014 in the Pediatric Gastroenterology outpatient clinic, and 100 patients were analyzed since four patients could not complete the study during the follow-up period. The exclusion criteria were: (1) Use of antibiotics, synbiotics, or PPIs during the last four weeks, (2) known allergy to antibiotics or synbiotics, (3) prior *H. pylori* treatment, and (4) recent GIS surgery.

### Diagnosis of *H. pylori*

All upper GIS endoscopies were performed by the same gastroenterologist using Olympus fiber endoscope Olympus Q260 or Olympus GIF P30 (Olympus, Tokyo, Japan). *H. pylori* was diagnosed based on histopathologi-cal assessment according to the Sydney classification of corpus and antrum biopsy samples (two samples from each) obtained from the upper inflamed gastric mucosa and fixed by 10% formalin, considering that the nodularities observed in the stomach in patients who underwent GIS endoscopy would support the diagnosis of *H. pylori.*

## EVALUATION OF THE PATIENTS

For each patient included in the study, a follow-up form was completed that contained the identity of the patient and contact details, date of birth, date of examination, fulfillment of the inclusion/exclusion criteria, body weight, height, and percentiles. The complaints on admission, such as dyspeptic complaints, such as flatulence or severe epigastric pain, reflux symptoms, such as nausea, vomiting or pyrosis, growth retardation, malabsorption findings, and chronic diarrhea were identified. Following endoscopy, the patients were randomized in order of delivering their results, and divided into two equal groups.

Informed consent forms were obtained after being read and signed by the patients’ families or themselves if they are older than 12 years, according to the treatment groups they were assigned.

For the study, approvals from the Şisfi Hamidiye Etfal Education and Research Hospital Ethics Committees and Turkish Medicines and Medical Devices Agency were sought and obtained.

## TREATMENT REGIMEN AND EVALUATION OF SYMPTOMS

Fifty patients who constituted group I received amoxi-cillin 50 mg/kg/day - 2 doses [Largopen® tablet, Bilim Pharmaceuticals, Tekirdag, Turkey] for 7 days, clarithro-mycin [Klacid®, Abbott laboratories, Dublin, Ireland] 15 mg/kg/day - 2 doses for 14 days, lansoprazole [Lansor® pellet capsule, Sanovel Pharmaceuticals, Istanbul, Turkey] 1 mg/kg/day - single dose, in the morning on an empty stomach for 14 days, and a synbiotic containing *B. lactis* (Maflor®) sachet - single dose for 14 days.

Fifty patients who constituted group II received the conventional triple therapy, i.e., amoxicillin 50 mg/kg/day - 2 doses for 14 days, clarithromycin 15 mg/kg/day - 2 doses for 14 days, and lansoprazole 1 mg/kg/day - single dose, in the morning on an empty stomach for 14 days.

All patients were given a detailed chart showing the methods of administration, durations of use and amounts of the drugs, and diaries for completion by them or their parents to note the compliance, symptoms, and side effects (such as epigastric pain, nausea and vomiting, bad metallic taste in the mouth, or diarrhea). These diaries were assessed by calling the patients within the first week of treatment, during an outpatient clinic visit at the end of treatment. Both groups were asked to return to the clinic for follow-up 1 month after the end of treatment and clinically evaluated in terms of side effects and symptoms. Antigen against *H. pylori* was tested in feces. Negative results were considered to be indicative of eradication of *H. pylori.*

## STATISTICAL EVALUATION

The statistical evaluation was performed using Statistical Package for the Social Sciences (SPSS) version 22.0. Mean, standard deviation, median, ratio, and frequency values were used for the descriptive statistics of the data. The distribution of the variables was controlled using Kolmogorov-Smirnov test. Independent samples t-test and Mann-Whitney U test were used to analyze the quantitative data. For the analysis of the qualitative data, chi-square test or, if the conditions of chi-square are not met, Fisher’s exact test was used. The results were evaluated at a confidence interval (CI) of 95%, and p < 0.05 was considered significant.

## OUTCOME MEASURES

### Evaluation of Treatment Success

Treatment was considered successful if the patient’s feces test became negative.

### Evaluation of Compliance and Side Effects

All patients were given diaries for completion by them or their parents to note the compliance, symptoms, and side effects.

## RESULTS

This study originally included 104 pediatric patients, but 3 of them were subsequently excluded due to the use of PPIs within the last 4 weeks and 1 was excluded due to antibiotic allergy, so the remaining 100 patients were divided into two groups by simple randomization method. The demographic data of the patients included in the study are shown in Table 1. Both groups completed the treatment period and the 1-month posttreat-ment period. No side effects requiring discontinuation were observed during treatment. There was no difference in gender or mean age between the groups (p = 0.841, p = 0.893).

**Table 1:** **Table 1:** Demographic data of the patients included in the study

				*Group I*								*Group II*									
				*Mean ± SD/n %*				*Med*		*Min-Max*		*Mean ± SD/n %*				*Med*		*Min-Max*		*p-value*	
Age				12.7 ± 3.3				13		6-17		11.4 ± 3.5				12		5-17		0.070	
Gender		Female		27		54.0%						28		56.0%						0.841	
		Male		23		46.0%						22		44.0%							

Independent samples t-test/chi-square test

**Flow Chart 1:**
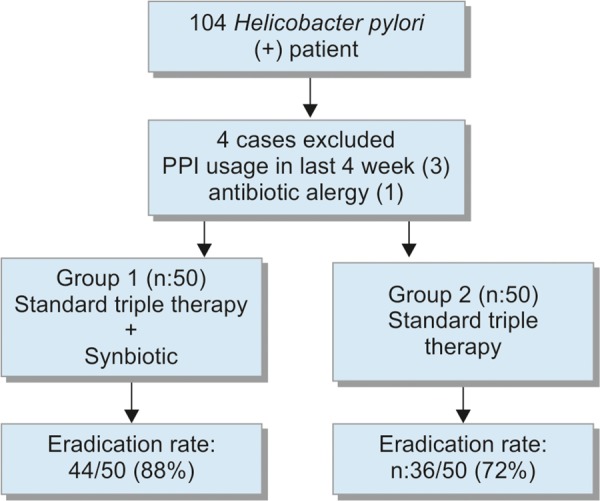
Numbers of patients enrolled in study

**Graph 1:**
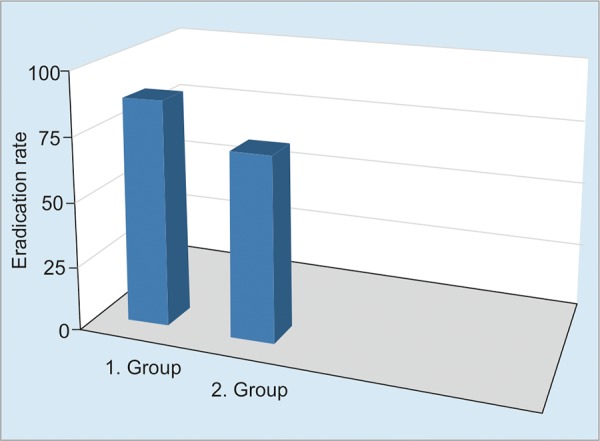
Posttreatment eradication rates in the groups (p = 0.046)

**Graph 2:**
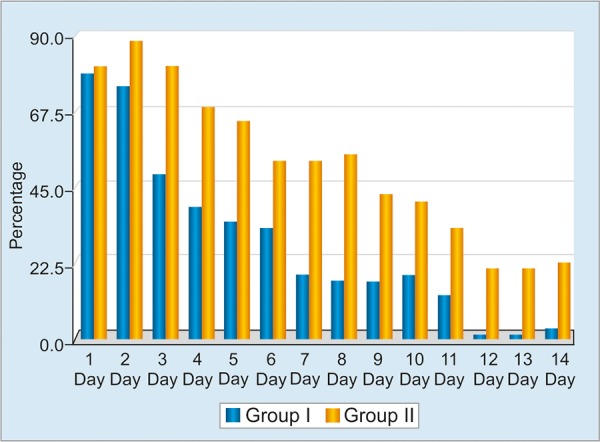
Occurrence rates of abdominal pain as a side effect during treatment in both groups

**Graph 3:**
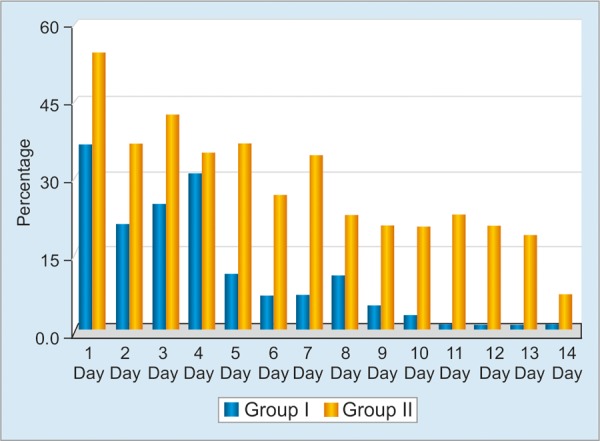
Occurrence rates of nausea as a side effect during treatment in both groups

The workflow of the study is summarized in Flow Chart 1. All patients were tested for antigen against *H. pylori* in feces 1 month after the end of the eradication treatment. Eradication of *H. pylori* was achieved in 44 of 50 patients (88%) in group I, and 36 of 50 patients (72%) in group II (Graph 1). The eradication rate was statistically higher in group I (p = 0.046, 95% CI: 2.85; 1.00-8.17). With respect to the side effects during treatment, the abdominal pain rates were not significantly different in groups I and II on days 1 and 2 (p > 0.05), while being significantly higher in group II between days 3 and 14 (p < 0.05) (Graph 2). The nausea and diarrhea rates were significantly lower in the group that received the probiotic (p < 0.05, p < 0.04) (Graph 3).

The rates of occurrence of vomiting on any day between days 1 and 7 were not significantly different in groups I and II (p = 0.86). The rate of occurrence of vomiting on any day between days 8 and 14 was significantly higher in group II than in group I (p < 0.027). The rates of occurrence of metallic taste were not significantly different between the groups (p = 0.418).

## DISCUSSION

Two antibiotics (amoxicillin + clarithromycin or metroni-dazole) and a PPI are used in the standard *H. pylori* eradication treatment in symptomatic children. ESPGHAN and NASPGHAN have recently recommended triple therapy with bismuth or sequential therapy as first-line regimen. Success rate in terms of eradication is still low, despite the recommended treatment regimen, and increased antibiotic resistance rates and low compliance to treatment have been implicated.^4,10,11^

Probiotics have been one of the alternative treatments used in an effort to increase eradication rates and to reduce side effect rates. Some probiotic strains have been found to be effective in increasing *H. pylori* eradication rates and reducing side effect rates, thereby increasing compliance. The Maastricht IV/Florence consensus report states that some prebiotics and probi-otics are promising to reduce side effects as an adjuvant therapy.^12^

In the literature, probiotics were either used alone or added to the standard therapy in studies on *H. pylori* and probiotics. Our study aimed to investigate the effect of synbiotic sachet added to the standard therapy on the eradication of *H. pylori* and on the side effect occurrence rates, which are the outcome measures used in the studies in the literature.

In terms of *H. pylori* eradication, our study demonstrated a significant increase in the group that received the synbiotic compared with the group without synbi-otic [(p = 0.046) 95% CI: 2.85 (1.00-8.17)]. The systematic reviews and meta-analyses on this subject show that the mostly studied probiotics are different species of *Lactobacilli* and *S. boulardii.* One of the studies reporting that probiotics increase the *H. pylori* eradication rates when added as adjuvants is the systematic review conducted by Szajewska et al.^13^ *S. boulardii* (500-1,000 mg 2-4 weeks) was added to the standard triple therapy and increased the *H. pylori* eradication rate compared with the control groups [relative risk (RR) = 1.13, 95% CI: 1.05-1.21]. Although the studies conducted by Song et al^14^ and Zojaji et al^15^ did not support this outcome, *S. boulardii* was stated as a promising adjuvant therapy.^11^ In the study conducted by Wang and Huang,^16^ *L. acidophilus* and *Bifidobacterium bifidum* were added to the standard triple therapy for 6 weeks, and the increase in the *H. pylori* eradication was considered significant compared with the group that did not receive *L. acidophilus* and *B. bifidum.* The meta-analysis conducted by Tong et al^17^ evaluated different probiotics, and found that 8 of 14 randomized controlled studies (RCSs) used a single probiotic strain and *Helicobacter* eradication rates increased in two studies that used *Lactobacilli.* When all results were evaluated together, the increase (84%) in the eradication rates in the group with probiotics was considered significant (RR 2.09, 95% CI 1.28-3.41).

In two studies that were conducted with addition of commercially available yoghurts containing *Bifidobacte-rium animalis* and *Lactobacillus casei* to the standard triple therapy, Sheu et al^18^ reported high eradication rates, while the study by Goldman et al^19^ found no difference in terms of eradication.

In contrast to our study, there are studies in the literature conducted with the same strain and found no significant difference in *H. pylori* eradication. The placebo-controlled study by Islek et al^20^ with the same strain found no significant difference in eradication (intent-to-treat, 80.8% and 67.3%, p = 0.13 respectively; per-protocol, 86.3% and 81.5%, p = 0.55 respectively). These authors gave the probiotic dose two times a day for 7 days. Akcam et al^21^ used *L. casei, L. acidophilus,* and *B. lactis* 2211 in addition to the standard therapy twice a day for 14 days. No significant difference was found between the two groups in terms of eradication (p = 0.78).

Studies conducted with LGG and *L. reuteri* and that demonstrated no difference in the eradication rates were also identified.^22,23^ Since it was stated that the increase in the *H. pylori* eradication may be strain-specific and RCSs with different species are warranted, we found that the eradication rate is significantly higher in our RCS in contrast to the studies conducted with this strain.

In our study, when the effect of the synbiotic added to the therapy on side effects was the second outcome measure, favorable effects were observed especially in terms of abdominal pain, nausea, vomiting, and diarrhea in the group that used the synbiotic. No significant difference was found in terms of dysgeusia. In the systematic review by Szajewska et al,^13^ *S. boulardii* was found favorable, especially in terms of diarrhea. The meta-analysis by Tong et al^17^ showed that probi-otics reduced the frequency of treatment-associated side effects with significantly lower occurrence rates, especially for diarrhea and dysgeusia. Akcam et al^21^ found no significant difference in terms of side effects either. In their study with the same strain, Islek et al^20^ found a significant reduction in side effect occurrence rates in the group that used the synbiotic compared with the placebo group (63% and 17% respectively, p < 0.01). In an Italian RC study, *L. reuterii* ATCC 55730 was added to 10-day sequential therapy; the severity of symptoms was assessed according to the gastrointestinal symptom rating scale, and a significant reduction in the occurrence rates of side effects was compared with placebo.^22,24^ The probiotic strains *S. boulardii, L. reuteri,* and LGG were reported to especially reduce diarrhea, which is one of the antibiotic-related side effects.^11^

The limitations of our study were the lack of placebo control, the lack of performance of *H. pylori* cultures and antibiotic sensitivity or resistance tests, and the lack of evaluation of the severity of symptoms. However, the side effects observed during treatment were evaluated through diaries on individual days, and we think that the evaluation of side effects by phone calls during the first week and in the clinic in the second week may have increased the compliance and positively affected the increase in eradication.

In contrast to the two studies with similar strains, our study demonstrated that the synbiotic added to the *H. pylori* treatment increases the eradication rate, significantly reduces the side effects of abdominal pain after day 3 and vomiting after week 2, and also has significant favorable effects on the occurrence rates of the side effects of diarrhea and nausea.

## CONCLUSION

In this study, the addition of synbiotic to the triple therapy is effective for eradicating *H. pylori* infection in children and usually is helpful to reduce or eliminate dyspeptic symptoms like abdominal pain, diarrhea, and vomiting. Performance of more studies on this subject is important to determine clear, evidence-based recommendations in the treatment approach.
